# Real-life prevalence of progressive fibrosing interstitial lung diseases

**DOI:** 10.1038/s41598-021-03481-8

**Published:** 2021-12-14

**Authors:** Maureen Gagliardi, Damienne Vande Berg, Charles-Edouard Heylen, Sandra Koenig, Delphine Hoton, Farah Tamirou, Thierry Pieters, Benoit Ghaye, Antoine Froidure

**Affiliations:** 1 Department of Pulmonology, Cliniques Universitaires Saint-Luc, Université Catholique de Louvain, Avenue Hippocrate, 10, 1200 Bruxelles, Belgium; 2 Department of Radiology, Cliniques Universitaires Saint-Luc, Université Catholique de Louvain, Bruxelles, Belgium; 3 Department of Pathology, Cliniques Universitaires Saint-Luc, Université Catholique de Louvain, Bruxelles, Belgium; 4 Department of Rheumatology, Cliniques Universitaires Saint-Luc, Université Catholique de Louvain, Bruxelles, Belgium

**Keywords:** Risk factors, Outcomes research

## Abstract

The concept of progressive fibrosing interstitial lung disease (PF-ILD) has recently emerged. However, real-life proportion of PF-ILDs outside IPF is still hard to evaluate. Therefore, we sought to estimate the proportion of PF-ILD in our ILD cohort. We also determined the proportion of ILD subtypes within PF-ILD and investigated factors associated with PF-ILDs. Finally, we quantified interobserver agreement between radiologists for the assessment of fibrosis. We reviewed the files of ILD patients discussed in multidisciplinary discussion between January 1st 2017 and December 31st 2019. Clinical data, pulmonary function tests (PFTs) and high-resolution computed tomography (HRCTs) were centrally reviewed. Fibrosis was defined as the presence of traction bronchiectasis, reticulations with/out honeycombing. Progression was defined as a relative forced vital capacity (FVC) decline of ≥ 10% in ≤ 24 months or 5% < FVC decline < 10% and progression of fibrosis on HRCT in ≤ 24 months. 464 consecutive ILD patients were included. 105 had a diagnosis of IPF (23%). Most frequent non-IPF ILD were connective tissue disease (CTD)-associated ILD (22%), hypersensitivity pneumonitis (13%), unclassifiable ILD (10%) and sarcoidosis (8%). Features of fibrosis were common (82% of CTD-ILD, 81% of HP, 95% of uILD). After review of HRCTs and PFTs, 68 patients (19% of non-IPF ILD) had a PF-ILD according to our criteria. Interobserver agreement for fibrosis between radiologists was excellent (Cohen’s kappa 0.86). The main diagnosis among PF-ILD were CTD-ILD (36%), HP (22%) and uILD (20%). PF-ILD patients were significantly older than non-F-ILD (P = 0.0005). PF-ILDs represent about 20% of ILDs outside IPF. This provides an estimation of the proportion of patients who might benefit from antifibrotics. Interobserver agreement between radiologists for the diagnosis of fibrotic ILD is excellent.

## Introduction

Interstitial lung diseases (ILDs) are a large heterogeneous group of diffuse parenchymal lung diseases. More than 200 entities have been described, calling for classification attempts. To date, ILDs are usually classified based on their underlying cause or condition: the official ATS-ERS classification from 2013^1^ divides ILDs into four subgroups, namely ILDs of known cause (related to an underlying disease, occupational, toxic etc.), granulomatous diseases, idiopathic ILDs and a last subgroup with orphan diseases (Langerhans cell histiocytosis, lymphangioleiomyomatosis etc.). Although such a classification is useful on a nosological point of view, it does not really capture the clinical expression of the disease, nor the underlying mechanisms. Therefore, the classification does not provide hints for a potential response to any given treatment. Conversely, it is possible to consider ILD based on their behaviour, either inflammatory, fibrotic, or mixed. At one end of the spectrum, inflammatory ILDs like cryptogenic organizing pneumonia (COP) usually respond well to immunosuppressive drugs^2^. However, immunosuppressant are deleterious in idiopathic pulmonary fibrosis (IPF)^3^and this disease represents the paradigm of a progressive and fibrotic ILD. Antifibrotic have emerged as an effective pharmacotherapy for the treatment of IPF since 2014^4,5^: pirfenidone and nintedanib can slow down lung function decline and prevent exacerbations. Furthermore, recent real-life data provided evidence for a prolonged survival of IPF patients treated with antifibrotics^6^. Beyond IPF, there is now a burden of evidence that other ILD also display signs of fibrosis and share common pathophysiological pathways with IPF^7–9^, paving the way for the use of antifibrotics outside IPF. In this context, the concept of progressive and fibrotic ILD (PF-ILD) emerged, suggesting that rather than splitting ILDs in multiple subcategories, it would be relevant to lump them based on their inflammatory or fibrotic aspect^10,11^. In line with this concept, recent clinical trials have demonstrated the efficacy of antifibrotics in non-IPF ILD: nintedanib can prevent lung function decline in systemic sclerosis-associated ILD^12^ and in progressive fibrosing ILD of various causes^13^. The effect of pirfenidone was evaluated in patients with progressive unclassifiable ILD (uILD study)^14^ and non-IPF PF-ILD (RELIEF study)^15^; although the primary endpoint was not met in neither both studies, subgroup analysis suggests that this drug might be beneficial for selected patients. Based on these encouraging results, a substantial proportion of ILD patients could benefit from antifibrotic drugs in the future. However, at this point, the actual proportion of the progressive and fibrosing phenotype within non-IPF ILD patients remains elusive. Recent studies set up in France and UK estimated that about 25% of ILD have a progressive and fibrosing phenotype^16–18^. The results of a recent survey conducted among pulmonologists, rheumatologists and internists^19^ suggest that up to 30% of ILD patients display signs of progression and fibrosis, but the design of the study did not allow for a central review of fibrosis nor progression. Finally, a recent retrospective study conducted in 120 non-IPF patients suggested that up to 68% fulfilled criteria for progression based on the inclusion criteria of the recent INBUILD, RELIEF and uILD study^20^. Therefore, we wished to evaluate the real-life proportion of PF-ILD within a large cohort of ILD patients treated according to current practice. We centrally reviewed all files, pulmonary function tests (PFTs) and high-resolution computed tomography (HRCTs) of consecutive patients discussed in multidisciplinary discussion (MDD) in a tertiary centre between 2017 and 2020.

## Materials and methods

### Study population

We included all ILD patients reviewed in multidisciplinary discussion (MDD) between January 1st 2017 and December 31st 2019, for whom HRCT and PFT data were available. During MDD, a panel composed of pulmonologists, radiologists, pathologists and rheumatologists analyses patients’ medical history, clinical, radiological, biological data as well as histological data when available. A final diagnosis is proposed if the team reaches an agreement. Every final MDD report is validated by the MDD coordinator (AF) and included in the patient’s electronic record, and we have previously published the details of our MDD process^21^. As idiopathic pulmonary fibrosis is a paradigm of a progressive and fibrosing disease, IPF patients were used as a comparator group. We classified non-IPF patients into nine diagnostic groups, namely connective-tissue disease-associated ILDs (CTD-ILDs), hypersensitivity pneumonitis (HP), unclassifiable ILD (uILDs), sarcoidosis, obstructive or constrictive bronchiolitis and (cryptogenic) organizing pneumonia (OB/OP), smoking-related diseases, drug-induced diseases, non-CTD systemic diseases and others. We collected all relevant demographic and clinical data.

We applied the STROBE criteria for observational studies (http://www.equator-network.org/wp-content/uploads/2015/10/STROBE_checklist_v4_combined.pdf).

### Pulmonary function tests and high-resolution computed tomography analysis

All pulmonary function tests (PFTs) available for patients were centrally reviewed. PFTs were performed according to the recommendations of the Global Lung Function Initiative (GLI).

We reviewed all chest CT from non-IPF ILD patients to assess the presence of fibrosis. A total of 308 chest HRCTs from the 291 non-IPF patients were reviewed. The vast majority (n = 256) of chest CT were performed with a 256-detector row scanner (ICT, Philips Healthcare, Cleveland, OH). Patients underwent standardized HRCT with the following parameters: collimation 256 × 0.625 mm, rotation time of 0.5 s, 100–120 kVp, 40 to 120 mA. One-mm thick contiguous or overlapping axial images were reconstructed on a 512 × 512 matrix. Remaining CTs (n = 52) were obtained from various centres with 1-mm (n = 29) or 3-mm (n = 23) thick axial slices. The quality of HRCT was evaluated as diagnostic in all patients. Two blinded junior thoracic radiologists (DV and CEH with 4 and 6-year experience in evaluating chest CT, respectively) analysed all HRCT from patients to determine the presence of fibrosis, defined by the presence of reticulations, traction bronchiectasis and/or honeycombing on dedicated workstations and screens (Radiforce RX250, EIZO Corporation, Hakusan, Ishikawa, Japan). Radiologists also provided a quantification of fibrosis (either less or at least 10 percent of lung parenchyma). Radiologists then provided a final assessment of fibrosis and an estimation of its extent (either less or at least ten percent of the lung parenchyma) during an adjudication session with a third radiologist having 20-year experience in thoracic imaging (BG).

### Endpoints

We sought to determine the proportion of non-IPF patients presenting with a progressive and fibrosing phenotype, consisting of both the presence of fibrosis on HRCT, defined as the combination of reticulations and traction bronchi(ol)ectasis, and evidence of disease progression.

We defined progression as a relative decline of at least 10 percent of forced vital capacity (FVC) or a relative FVC decline of 5 to 10 percent along with progression of lesions on HRCT within maximum 24 months.

We also wished to assess the interobserver agreement between radiologists on the presence of fibrosis and the quantification of fibrotic lesions (more or less than ten percent).

### Statistics

We used one-way ANOVA followed by Holm-Sidak’s multiple comparisons test for comparison between groups. Contingency were analysed with Chi-Square test and Fisher’s exact test. Multiple survival curves were analysed with Log-Rank (Mantel-Cox) test. We quantified interobserver agreement with Cohen’s kappa coefficient.

All statistics were reviewed by the *Plateforme de support en méthodologie et calcul statistique (SMCS)*, UCLouvain.

### Ethical considerations

Patients’ informed consent was waived due to the retrospective aspect of the study. The present study was approved by our local ethics committee (study PNEU-ILD-02, approval number 2018/15MAR/116).

### Ethics approval and consent to participate

Patients’ informed consent was waived due to the retrospective aspect of the study. The present study was approved by our local ethics committee (Comité d’éthique Hospitalo-facultaire des Cliniques universtitaires Saint-Luc, study PNEU-ILD-02, approval number 2018/15MAR/116).

The authors conducted this research in full accordance with the Declaration of Helsinki.

### Consent for publication

See the “Ethical considerations” section of the “Materials and methods” section.

## Results

### Patients’ characteristics

Between January 1st 2017 and December 31st 2019, 490 patients were discussed in MDD. Twenty-six patients were withdrawn from the analysis as access to their HRCT pictures was not granted for central review. Thus, we included 464 patients in the analysis. A confident diagnosis of IPF was made for 105 patients (76 men, 29 women, representing 23% of total ILDs), of whom 91 (87%) were treated with an antifibrotic drug (44 received pirfenidone, 47 received nintedanib). All 359 remaining non-IPF patients were centrally assessed for fibrosis and progression. We provide a flowchart of the study population in Figure S1.

Main ILD patients’ characteristics are shown in Table 1.

**Table 1:** Patients’ characteristics.

	Idiopathic pulmonary fibrosis N = 105	Non-IPF ILD			P value
		PF-ILD N = 68	F-ILD N = 170	Non-F-ILD N = 121	
Age (years, median and range)	73 (58–92)	68 (36–91)	66 (24–89	53 (18–90)	P < 0.0001
Male gender (%)	72	38	54	35	P < 0.0001
Current or former smoker (%)	73	29	51	40	P < 0.0001
Immunosuppressive treatment (%)	NA	63	51	39	P = 0.14
Antifibrotics	Pirfenidone N = 44 Nintedanib N = 47	NA			

*NA* not applicable.

**Figure 1:**
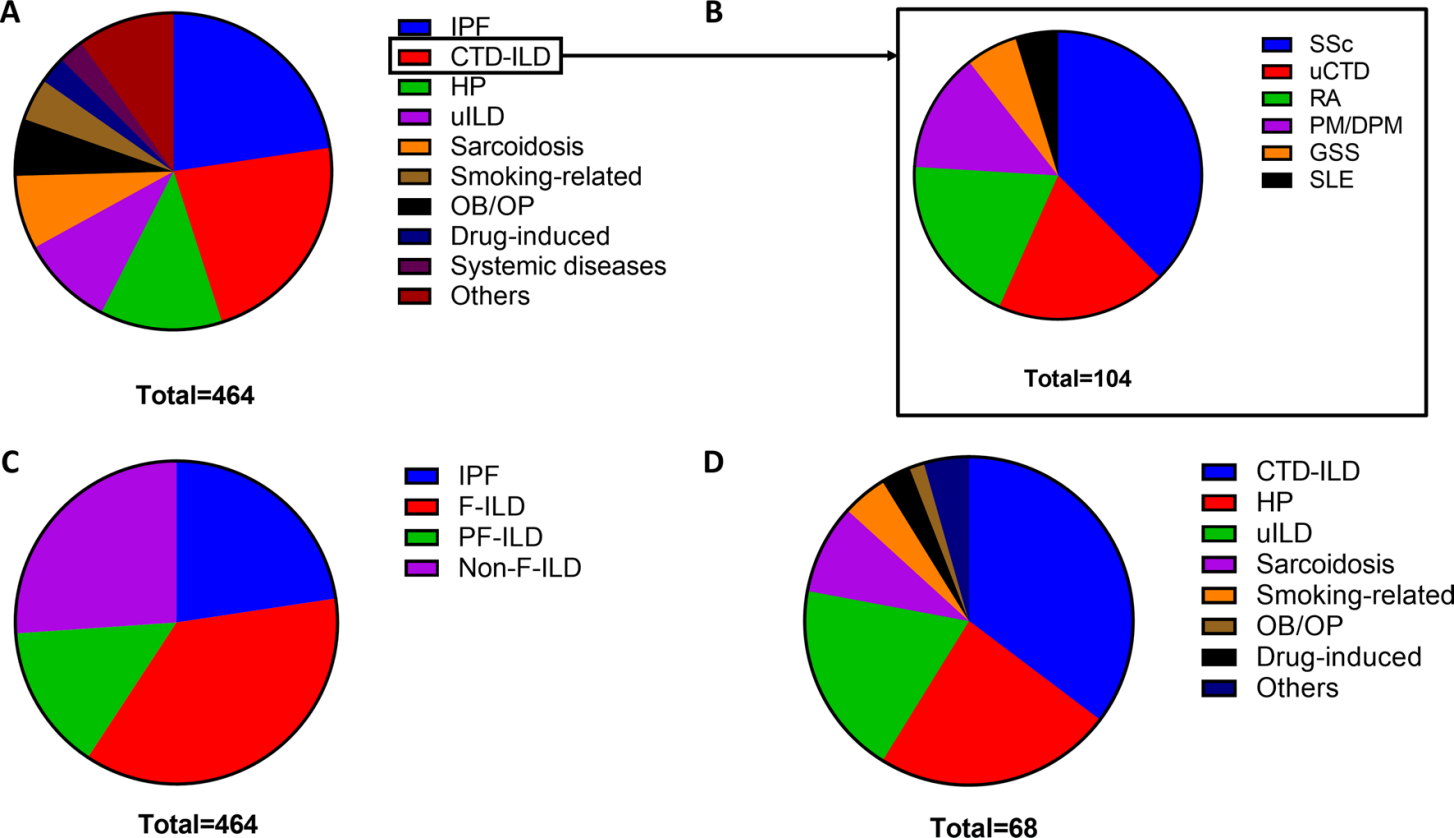
Relative proportion of ILD subtypes within all ILD (panel **A**). Panel (**B**) shows CTD subgroups within CTD-ILD. Panel (**C**) shows ILD subclasses (based on fibrosis, inflammation and progression) within the whole population and panel (**D**) the proportion of ILD within non-IPF PF-ILD.

After IPF (N = 105, 23%), the main diagnoses were connective tissue disease-related ILDs (CTD-ILDs, N = 104, 22%), hypersensitivity pneumonitis (N = 58, 13%), unclassifiable ILDs (N = 44, 10%) and sarcoidosis (N = 35, 8%) (Fig. 1A). CTD-ILDs were mainly related to systemic sclerosis (N = 39), undifferentiated CTD (N = 20) and rheumatoid arthritis (N = 20) (Fig. 1B).

Among non-IPF patients, 87 were treated with systemic corticosteroids alone, 110 received a combination of immunosuppressants and corticosteroids. The most prescribed immunosuppressive drugs were mycophenolate mofetyl (N = 42) and methotrexate (N = 26). Twenty-two HP patients benefitted from avoidance of the causal agent without pharmacological treatment, 86 non-IPF patients were simply followed, and 14 received a biological agent (monoclonal antibody). The remaining 30 received other treatments including inhaled corticosteroids (3 patients), sirolimus (3 patients), along with close follow-up. No patients were lost to follow-up.

### Proportion of fibrosing ILD and progressive fibrosing phenotype within ILD subtypes

**Figure 2:**
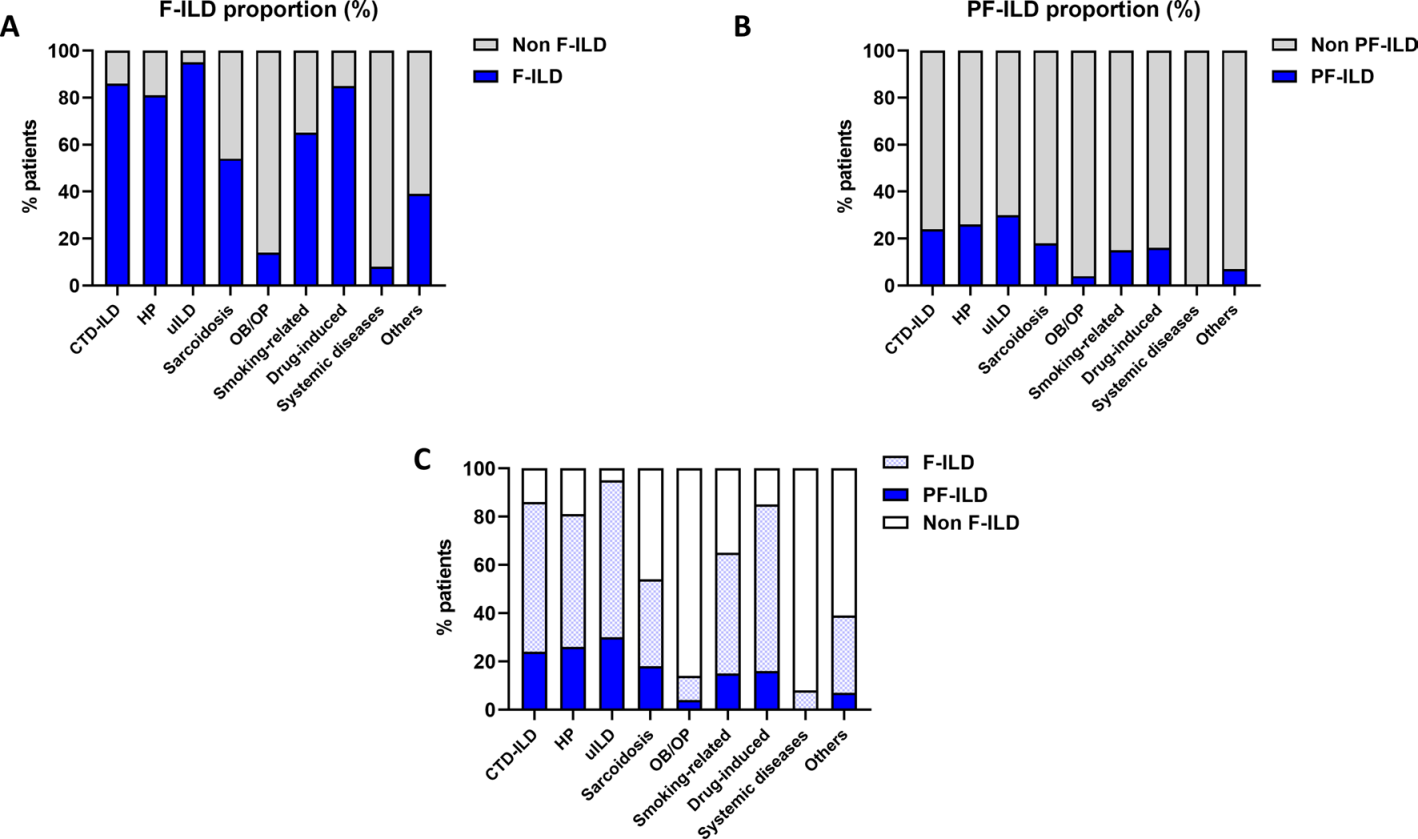
Proportion of fibrosing ILD (panel **A**), progressive and fibrosing ILD (panel **B**) and merge (panel **C**) within ILD subgroups.

We found evidence of fibrosis on HRCT in 240 patients (67%), confirmed at histology in 30 patients. Of note, only three biopsies were performed in non-fibrosing ILD patients. For 155 patients (43%), fibrosis affected at least 10% of lung parenchyma according to the radiologists’ analysis. When looking into prespecified ILD subgroups, fibrotic lesions were identified in 86% of CTD-ILDs, 81% of HP, 95% of uILDs, 54% of sarcoidosis, 14% of OB/OP, 65% of smoking-related diseases, 85% of drug-induced ILDs, 8% of ILDs related to non-CTD systemic diseases and 39% of the other ILD (Fig. 2A).

**Figure 3:**
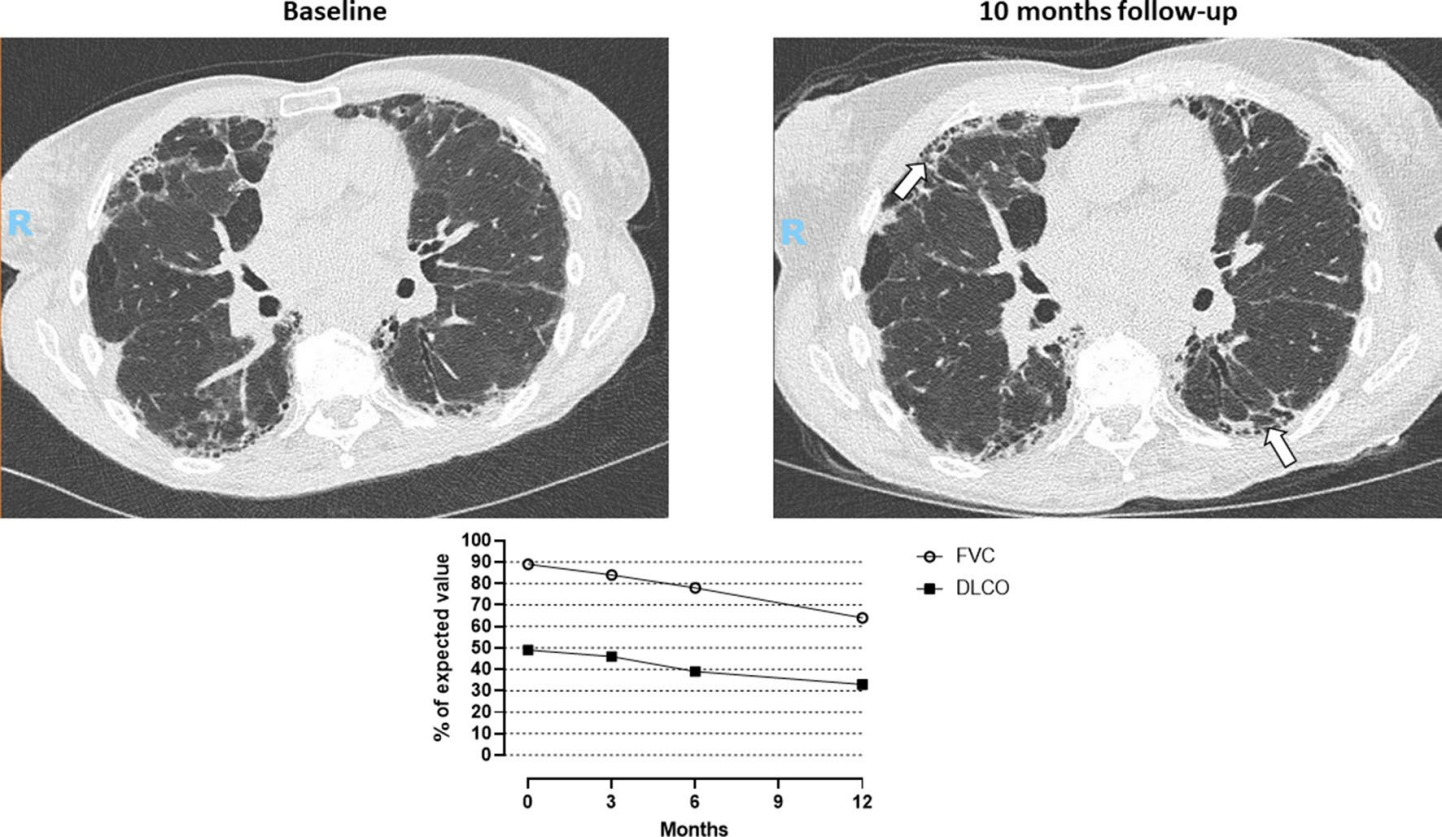
Example of a patient suffering from fibrosing hypersensitivity pneumonitis. 10 month follow-up high-resolution CT scan shows progression of fibrosis with honeycombing (white arrows). Longitudinal pulmonary function tests demonstrate decline of both forced vital capacity and lung diffusion capacity. FVC: forced vital capacity; DLCO: lung diffusion capacity.

We then combined analysis of sequential HRCT and PFTs to identify patients with a PF-ILD: criteria for PF-ILD were met in 68 patients (19% of all non-IPF ILD). The main diagnoses were CTD-ILD (N = 24), HP (N = 16) and uILD (N = 13) (Fig. 1C,D). Median relative FVC decline of PF-ILD patients was 13% (range 5–40) within a median interval of 387 days (range 14–717). In our study population, PF-ILDs represented 24% of CTD-ILD, 26% of HP, 30% of uILD, 18% of sarcoidosis, 4% of OB/OP, 15% of smoking-related diseases, 16% of drug-induced ILD, zero percent of ILD related to non-CTD systemic diseases and seven percent of the other ILD (Fig. 2B,C). An example of a PF-ILD patient (HRCT and PFT evolution) is provided in Fig. 3.

### Factors associated with the progressive fibrosing phenotype

**Figure 4:**
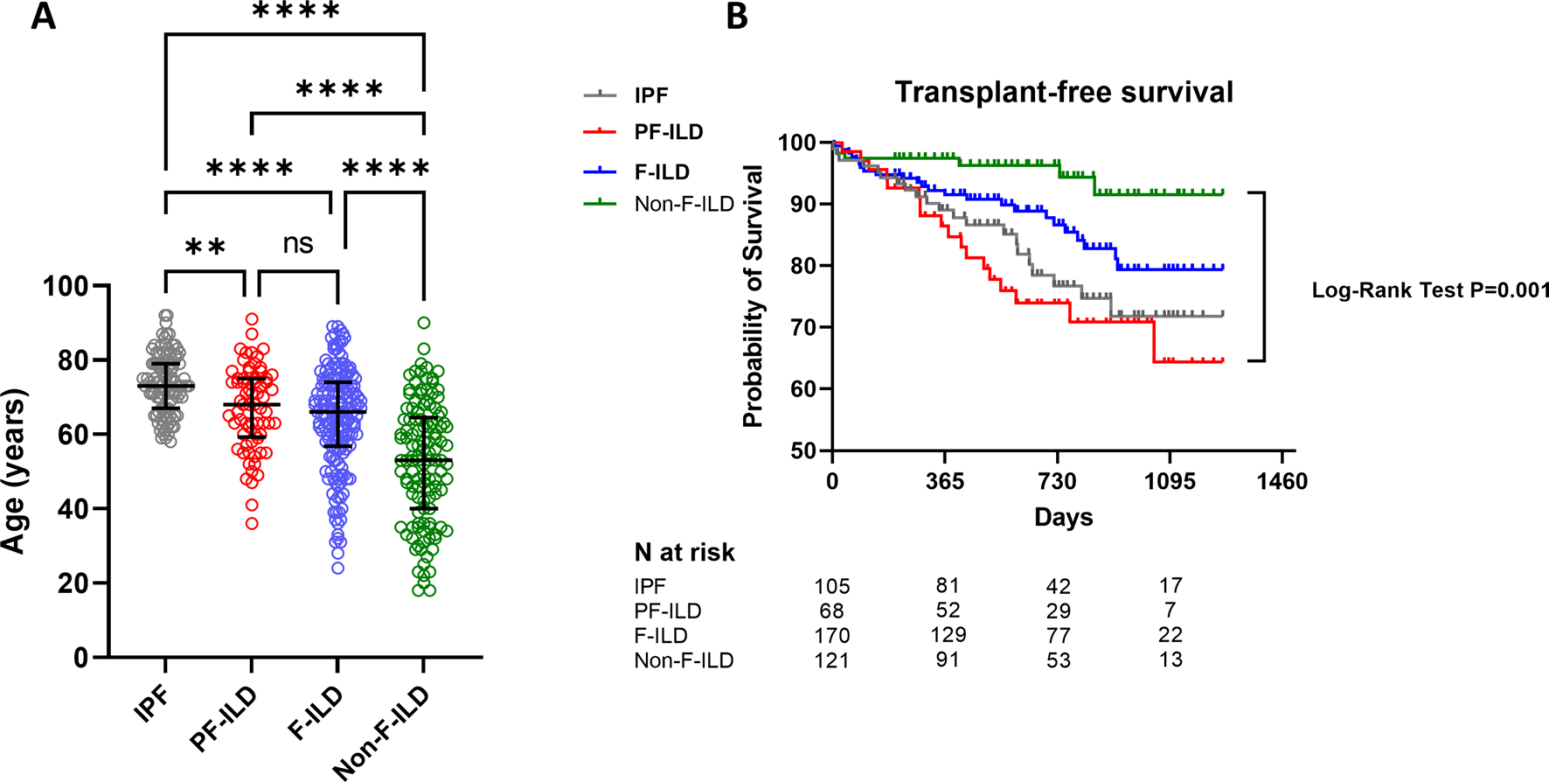
Clinical factors associated with ILD. Panel A shows a significant difference in age between IPF, PF-ILD/F-ILD and non-F-ILD (statistics, ANOVA followed by Holm-Sidak’s multiple comparisons test. Panel B shows transplant-free survival of IPF (grey line), PF-ILD (red line) and non-F-ILD (blue line) and Non-F-ILD (green). Statistics: Log-Rank test.

We compared the 68 patients with a non-IPF-PF-ILD with the rest of the study population (IPF, N = 105 F-ILD without progression, N = 170 and non-fibrosing-ILD, N = 121). As compared to the rest of the population, IPF patients were significantly older and were more frequently men and current or ex-smokers (Chi-Square test P < 0.0001, Fig. 4A). However, within non-IPF ILD, we did not find any association between PF-ILD and gender, smoking status, or underlying treatment (Table 1). Non-IPF PF-ILD and F-ILD patients were significantly older than non-F-ILD (P = 0.0001) (Fig. 4A). However, Among non-IPF fibrotic diseases, age was not associated with progression (P = 0.14).

Finally, we compared survival of IPF patients with our PF-ILD, F-ILD and non-F-ILD groups. As shown in Fig. 4B, non-F-ILD had a significantly better survival as compared to PF-ILD, F-ILD and IPF. Three-year survival was 91% for non-F-ILD, 79% for F-ILD, 72% for IPF and 64% for PF-ILD (Log-Rank test, P = 0.002). Median follow-up was 598 days for IPF, 651 days for non-IPF PF-ILD and 1106 days for non-PF-ILD.

### Interobserver agreement for assessment of fibrosis

When considering the consensus session with the expert radiologist as our gold standard, reader 1 and reader 2 had a sensitivity for fibrosis assessment of respectively 98.0% and 99.2%. Their respective specificity was 89.3% and 98%. Agreement between both readers and the consensus session was very high (Cohen’s kappa 0.879 for reader 1, 0.967 between reader 2, P = 0.0001).

Interobserver agreement between reader 1 and reader 2 was high (Cohen’s kappa 0.847, P = 0.0001, Table 2). We also assessed radiologists’ agreement for fibrosis quantification (below or more than 10% of the lung parenchyma). Compared to the consensus session, reader 1 had a sensitivity of 98.1% and a specificity of 90.7% for detection of fibrosis ≥ 10% of lung parenchyma; reader 2 had a sensitivity of 74.8% and a specificity of 98.0%. Agreement between both readers and the consensus session was high (Cohen’s kappa 0.888 for reader 1, 0.726 between reader 2, P = 0.0001). Interobserver agreement between reader 1 and reader 2 for determining fibrosis ≥ 10% of parenchyma was moderate to high (Cohen’s kappa 0.632, P = 0.0001, Table 2).

**Table 2:** Agreement between radiologists for fibrosis assessment.

Item	Interobserver agreement (Cohen’s Kappa)
Reticulations	0.634
Traction bronchiectasis	0.735
Honeycombing	0.720
Fibrosis	0.847
Fibrosis extend (< / ≥ 10%)	0.632

## Discussion

In this study, we evaluated the real-life prevalence of PF-ILD outside IPF in a large cohort of ILD patients, using objective criteria based on PFTs and HRCTs. The most frequent ILD diagnoses were IPF (23%), CTD-ILDs (22%), HP (13%) and uILDs (10%). We found features of fibrosis in a large proportion of patients. However, combined fibrosis and progression affected a lower, yet substantial, proportion of patients (19% of non-IPF patients). Patients with PF-ILD were significantly younger than IPF patients but older than non-F-ILD patients. When we analysed factors associated with PF-ILD outside of IPF, we did not find any association with ILD treatment, smoking status, and gender. Furthermore, PF-ILD and IPF survival were similar and significantly worse than F-ILD and non-F-ILD. Finally, our results showed a good to excellent interobserver agreement for fibrosis analysis on HRCT.

This study constitutes one of the first attempts to evaluate the real-life prevalence of the progressive and fibrosing phenotype of ILD using objective criteria of PFT decline and HRCT fibrotic features. Unlike the INBUILD inclusion criteria^13^, we did not use the notion of “clinical progression”. Indeed, the retrospective aspect of our work did not allow us to properly evaluate longitudinal clinical progression. This left us with PFTs and HRCT to evaluate fibrosis and progression. This point might have led to an underestimation of progression. However, data from IPF suggest a good correlation between FVC decline and disease worsening^22^. Furthermore, Outside IPF, PF-ILDs represented 19% of our population, in line with existing literature on the matter^17,18^. Analysis conducted in recent cohorts of ILD^23^ and in CTD patients led to similar results^24^. Main diseases associated with fibrosis and progression were CTD-ILDs, HP and uILDs. This reflects the proportion of patients included in the INBUILD study^25^. When we investigated clinical factors among non-IPF patients that would potentially be associated with PF-ILD, we did not find any association with gender, smoking status, or treatment. This tends to indicate that immunosuppressive drugs, although widely used for ILD, fail to prevent the development of a PF-ILD. This point was already suggested by a study conducted by De Sadeleer et al*.* among patients with chronic HP^26^. Interestingly, we found significant differences in age between IPF, PF-ILD and non-F-ILD. As IPF may be considered as the paradigm of a fibrotic lung disease while PF-ILD may combine fibrosis and inflammation, our finding reinforces the notion that ageing plays a crucial role in fibrosis development. Experimental studies showed that ageing cells display a stronger activation of pro-fibrotic pathways^27^. Mechanisms involved include telomeres dysfunction^28,29^ lack of regulation by B cells, a higher endoplasmic reticulum stress and an age-related shift in fibroblasts populations that precludes proper wound healing^30–32^. When we analysed survival, we found that IPF and PF-ILD patients had a significantly worse survival than F-ILD and non-F-ILD. This is also in line with the results of De Sadeleer et al*.*, that demonstrated a worse survival of fibrotic HP as compared to non-fibrotic HP^26^. Similarly, it was shown that fibrotic lung diseases related to mutations in telomerase-related genes had a poor survival regardless of their radiological or pathological characteristics^33^.

We also assessed the ability of radiologists to detect fibrosis by studying interobserver agreement of two junior thoracic radiologists (less than six-year experience) and an expert thoracic radiologist. We found excellent interobserver agreement for the detection of fibrosis and a good interobserver agreement for the quantification of fibrosis (either less or at least 10% of the parenchyma), which is much better than interobserver agreement for detecting a usual interstitial pneumonia (UIP) pattern^34^. Our results reflect the fact that low interobserver agreement for a UIP pattern is due to the difficulty to differentiate honeycombing and traction bronchiectasis^35^. This subtle difference was not considered in our criteria for fibrosis on HRCT. Of note, interobserver agreement was better for fibrosis assessment in general than for each feature of fibrosis taken separately. Although expected, these results illustrate that thoracic radiologists are quite concordant when it comes to evaluate the fibrotic aspect of HRCT in a patient, regardless the specific features present. This point also demonstrates that evaluation of fibrosis on HRCT is feasible for most thoracic radiologists, even with less than six-year experience.

Considering the current absence of an international consensus for the definition of the progressive and fibrosing phenotype, we suggest that our study provides a reliable estimate of the prevalence of PF-ILD in a large cohort of non-IPF patients, which gives an estimate of the proportion of patients who might benefit from antifibrotic drugs. We think that our results may help to guide future practice, as the current price of antifibrotics combined with limited health resources should warrant their use in well-selected patients. Of note, several studies evaluated the cost-effectiveness of nintedanib and pirfenidone in IPF^36,37^. Although they yielded conflicting results, the common agreement at this point is that antifibrotics may be considered cost-effective when used in properly selected patients^38^. The remaining uncertainties in the field of PF-ILD management warrant further research and collaboration between expert centres, as recently stated in two perspective articles^39,40^.

Of course, its retrospective aspect is a limitation of our work, and our data have possibly been influenced by baseline ILD treatments. However, we could not find a significant association between treatment (and especially immunosuppressive drugs) and the likelihood of progression. Differences between our cohort and the inclusion criteria of the INBUILD trial^13^ warrants caution when interpreting the potential role of antifibrotics for patients with PF-ILD. Furthermore, key questions remain unanswered. Our results did not allow us to analyse the slope of progression (in other words, whether progression is constant throughout time). Prospective studies in which serial PFTs are obtained within ILD subgroups could be conducted in the future. Additionally, whether data would have been modified in the absence of baseline treatment is also unknown. Finally, whether the age of patients influenced the treatment decisions at baseline is unknown.

## Conclusion

Our findings indicate that approximately one fifth of patients in a large ILD cohort display a progressive and fibrosing phenotype. In addition to providing information on the subset of patients who might benefit from treatment with antifibrotic drugs, we believe that our work paves the way for future investigations in the field of interstitial lung diseases.

## Supplementary Information

Supplementary Information 1.

## Data Availability

Our secured database contains several information used in other research projects and patients’ identifiers. Therefore, we cannot share it completely. However, if requested, we will consider providing raw data.

## Abbreviations

COP: Cryptogenic organizing pneumonia
CTD: Connective-tissue disease
FVC: Forced vital capacity
HP: Hypersensitivity pneumonitis
HRCT: High-resolution computed tomography
ILD: Interstitial lung disease
IPF: Idiopathic pulmonary fibrosis
MDD: Multidisciplinary discussion
PF-ILD: Progressive and fibrosing interstitial lung disease
PFT: Pulmonary function test
uILD: Unclassifiable ILD
