# Supplementary figures and images for: Real-life prevalence of progressive fibrosing interstitial lung diseases
Source: Sci Rep. 2021 Dec 14;11:23988. doi: 10.1038/s41598-021-03481-8 (PMC8671400; doi:10.1038/s41598-021-03481-8)

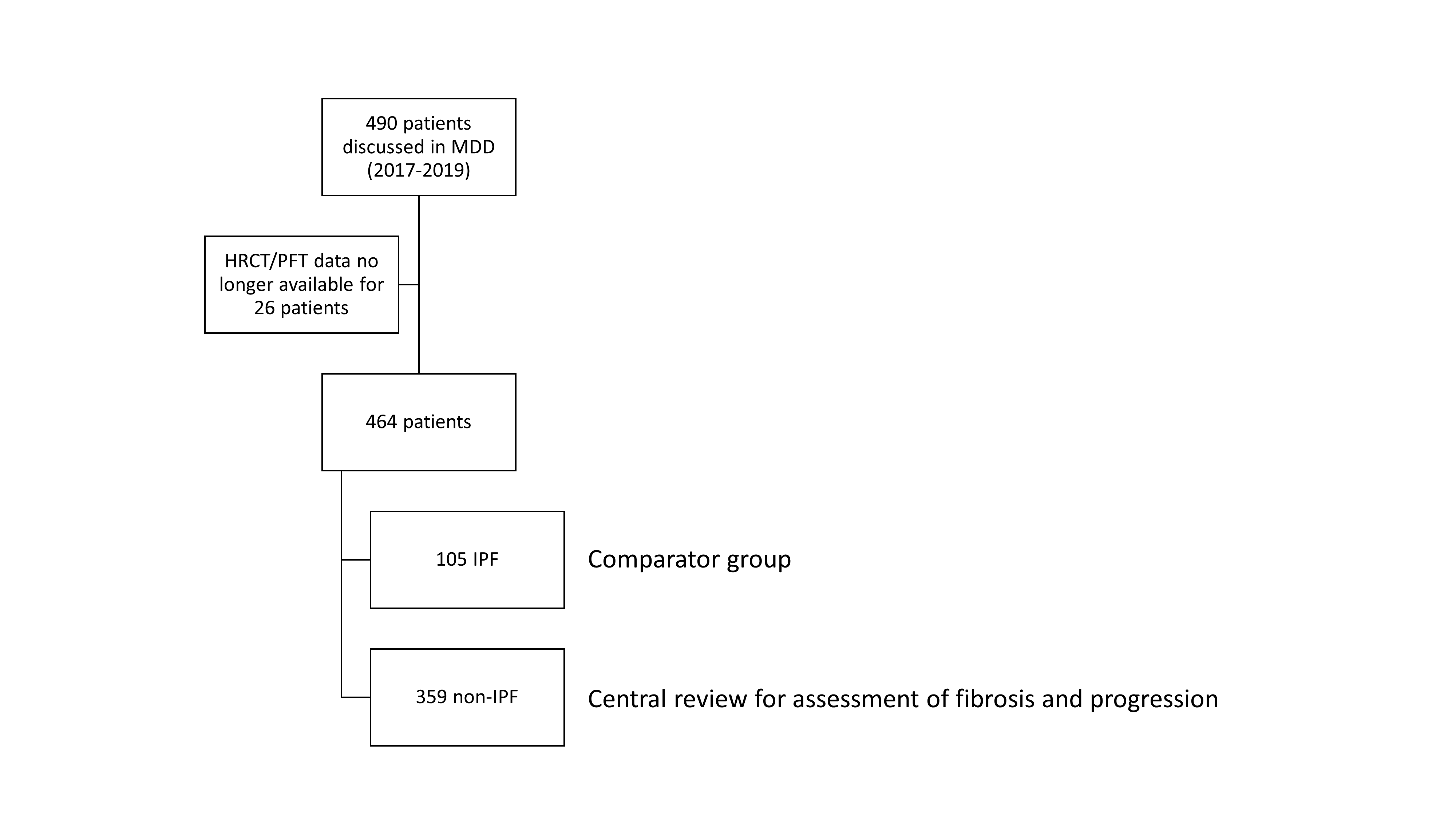

Supplement: Supplementary file 1 — Supplementary Information 1. [file 41598_2021_3481_MOESM1_ESM.tif]
